# Influence of Climate upon Consumption

**Published:** 1852-11

**Authors:** 


*Influence of Climate upon Consumption.*—The value of a removal to the south, of persons affected in the northern states with consumption, has been heretofore very generally admitted; but it is now asked whether much, if any, advantage is to be derived from spending merely the winter months at the south and returning to the north in the spring—and it is added that if a temperate atmosphere be all that is needed, this may be obtained in New England by means of a well-regulated system of artificial heat. We believe it to be an error to suppose that the southern states owe their immunity from phthisis pulmonalis alone to the mildness of their winters. If such were the fact, all temperate climates ought to be equally exempt, and all cold latitudes alike unfavorable. Yet phthisis is much more common upon the seaboard and in the mountainous districts of the southern states than at intermediate points, and it is comparatively rare in the northern portions of Canada and Russia, whilst it makes frightful havoc in milder England, France and our northern states.—*Southern Med. and Surg. Jour., Editorial.*

